# (1*E*,3*E*,5*E*,7*E*)-4,4′-(Octa-1,3,5,7-tetra­ene-1,8-di­yl)dipyridine

**DOI:** 10.1107/S160053681000317X

**Published:** 2010-02-03

**Authors:** Muhammad Nadeem Arshad, Mamoun M. Bader, Phuong-Truc T. Pham, K. Travis Holman

**Affiliations:** a Department of Chemistry, GC University Lahore 54000, Pakistan; b Department of Chemistry, Pennsylvania State University, Hazleton, PA 18202, USA; c Department of Chemistry, Pennsylvania State University, Worthington Scranton, PA 18512, USA; d Department of Chemistry, Georgetown University, 37th and O St. NW, Washington, DC 20057, USA

## Abstract

The title compound, C_18_H_16_N_2_, crystallizes with one and a half independent mol­ecules in the asymmetric unit, with the half-mol­ecule being completed by crystallographic inversion symmetry. Both independent mol­ecules are almost planar, with the non-H atoms exhibiting r.m.s. deviations from the least-squares mol­ecular plane of 0.175 and 0.118 Å, respectively.

## Related literature

For the synthesis, see: Woitellier *et al.* (1989 ▶). For the use of the diene and the triene in the synthesis of ladderanes *via* template-directed photochemistry, see: Gao *et al.* (2004 ▶). For a related structure, see: Bader (2009 ▶).
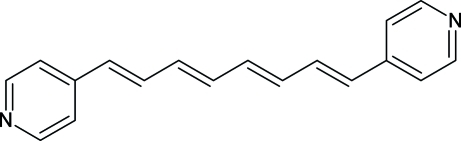


## Experimental

### 

#### Crystal data

C_18_H_16_N_2_
*M* *_r_* = 260.33
Monoclinic, 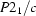

*a* = 5.5565 (12) Å
*b* = 17.950 (4) Å
*c* = 21.542 (5) Å
β = 94.809 (5)°
*V* = 2141.0 (8) Å^3^
*Z* = 6
Mo *K*α radiation
μ = 0.07 mm^−1^
*T* = 173 K
0.50 × 0.23 × 0.20 mm

#### Data collection

Siemens SMART 1K diffractometer
Absorption correction: multi-scan (*SADABS*; Bruker 2001 ▶) *T*_min_ = 0.979, *T*_max_ = 0.986
13659 measured reflections
4727 independent reflections
2195 reflections with *I* > 2σ(*I*)
*R*_int_ = 0.061

#### Refinement

*R*[*F* ^2^ > 2σ(*F* ^2^)] = 0.045
*wR*(*F* ^2^) = 0.102
*S* = 0.87
4727 reflections
343 parameters
H atoms treated by a mixture of independent and constrained refinement
Δρ_max_ = 0.15 e Å^−3^
Δρ_min_ = −0.17 e Å^−3^

### 

Data collection: *SMART* (Bruker, 2001 ▶); cell refinement: *SAINT* (Bruker, 2001 ▶); data reduction: *SAINT*; program(s) used to solve structure: *SHELXS97* (Sheldrick, 2008 ▶); program(s) used to refine structure: *SHELXL97* (Sheldrick, 2008 ▶); molecular graphics: *ORTEP-3 for Windows* (Farrugia, 1997 ▶), *PLATON* (Spek, 2009 ▶) and *X-SEED* (Barbour, 2001 ▶)’; software used to prepare material for publication: *WinGX* (Farrugia, 1999 ▶) and *PLATON*.

## Supplementary Material

Crystal structure: contains datablocks I, global. DOI: 10.1107/S160053681000317X/ng2706sup1.cif

Structure factors: contains datablocks I. DOI: 10.1107/S160053681000317X/ng2706Isup2.hkl

Additional supplementary materials:  crystallographic information; 3D view; checkCIF report

## Figures and Tables

**Fig. 1.**
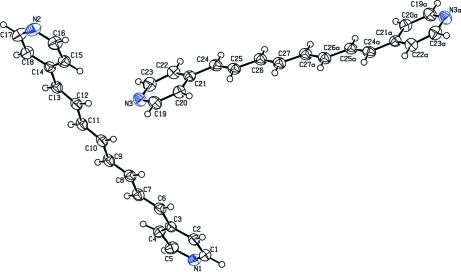
The labelled thermal ellipsoids plot with 50% probability level.

**Fig. 2.**
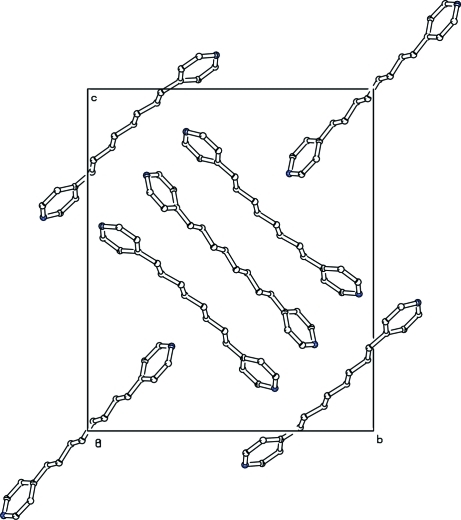
Unit cell packing for the title compound. Hydrogen atoms have been omitted for clarity.

**Fig. 3.**
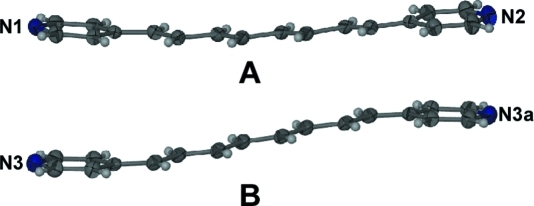
Planarity comparison for unique molecules A and B.

## supplementary crystallographic information

### Comment

The present bispyridyl tetraene is in continuation to our previously reported crystal structure of the bispyridyl triene analog (Bader, 2009). The diene and triene have been used in the synthesis of ladderanes *via* template directed photochemistry (Gao *et al.*, 2004). The electron transfer properties of metal complexes of bispyridyl polyenes have also been studied and the crystal structure of a metal complex of the title compound was reported (Woitellier *et al.*, 1989).

The title compound crystallizes in the monoclinic crystal system such that there are one and one-half molecules in the asymmetric unit, molecules A and B, respectively. Molecule B resides about an inverison center. The bond lengths of molecules A and B are comparable to the previously published structure of the triene derivative, namely 4-[(1E,3E,5E)-6-(4-pyridyl)hexa-1,3,5-trienyl]pyridine (Bader, 2009) and also the previously reported metal complex of the title compound (Woitellier *et al.*, 1989). Molecules A and B both deviate significantly from planarity (Fig. 3). The root mean square deviation of the carbon and nitrogen atoms from the least squares plane defined by such atoms in molecule A measures 0.175 Å, with N3 deviating from the plane by as much as 0.33 Å. The dihedral angle between the two planar pyridine rings of molecule A measures 7.53(0.11)°. Similarly, the root mean square deviation of the carbon and nitrogen atoms from the least squares plane defined by such atoms in molecule B measures 0.118 Å, with C25 being located 0.17 Å from the plane.

### Experimental

The compound was synthesized following the literature method (Woitellier *et al.*, 1989).

### Refinement

The C—H = 0.942–0.996 Å, H-atoms were located in difference map and refined: with *U*_iso_(H) = 1.2 *U*_eq_(C).

### Crystal data

**Table:**

C_18_H_16_N_2_	*F*(000) = 828
*M**_r_* = 260.33	*D*_x_ = 1.211 Mg m^−^^3^
Monoclinic, *P*2_1_/*c*	Mo *K*α radiation, λ = 0.71073 Å
Hall symbol: -P 2ybc	Cell parameters from 1908 reflections
*a* = 5.5565 (12) Å	θ = 2.3–24.0°
*b* = 17.950 (4) Å	µ = 0.07 mm^−^^1^
*c* = 21.542 (5) Å	*T* = 173 K
β = 94.809 (5)°	Needle, brown
*V* = 2141.0 (8) Å^3^	0.50 × 0.23 × 0.20 mm
*Z* = 6	

### Data collection

**Table:**

Siemens SMART 1K diffractometer	2195 reflections with *I* > 2σ(*I*)
ω scans	*R*_int_ = 0.061
Absorption correction: multi-scan (*SADABS*; Bruker 2001)	θ_max_ = 27.2°, θ_min_ = 1.9°
*T*_min_ = 0.979, *T*_max_ = 0.986	*h* = −5→7
13659 measured reflections	*k* = −20→23
4727 independent reflections	*l* = −27→21

### Refinement

**Table:**

Refinement on *F*^2^	Primary atom site location: structure-invariant direct methods
Least-squares matrix: full	Secondary atom site location: difference Fourier map
*R*[*F*^2^ > 2σ(*F*^2^)] = 0.045	Hydrogen site location: inferred from neighbouring sites
*wR*(*F*^2^) = 0.102	H atoms treated by a mixture of independent and constrained refinement
*S* = 0.87	*w* = 1/[σ^2^(*F*_o_^2^) + (0.0357*P*)^2^] where *P* = (*F*_o_^2^ + 2*F*_c_^2^)/3
4727 reflections	(Δ/σ)_max_ < 0.001
343 parameters	Δρ_max_ = 0.15 e Å^−^^3^
0 restraints	Δρ_min_ = −0.17 e Å^−^^3^

### Special details

**Table:**

Geometry. All s.u.'s (except the s.u. in the dihedral angle between two l.s. planes) are estimated using the full covariance matrix. The cell s.u.'s are taken into account individually in the estimation of s.u.'s in distances, angles and torsion angles; correlations between s.u.'s in cell parameters are only used when they are defined by crystal symmetry. An approximate (isotropic) treatment of cell s.u.'s is used for estimating s.u.'s involving l.s. planes.
Refinement. Refinement of *F*^2^ against ALL reflections. The weighted *R*-factor *wR* and goodness of fit *S* are based on *F*^2^, conventional *R*-factors *R* are based on *F*, with *F* set to zero for negative *F*^2^. The threshold expression of *F*^2^ > 2σ(*F*^2^) is used only for calculating *R*-factors(gt) *etc*. and is not relevant to the choice of reflections for refinement. *R*-factors based on *F*^2^ are statistically about twice as large as those based on *F*, and *R*- factors based on ALL data will be even larger.

### Fractional atomic coordinates and isotropic or equivalent isotropic displacement parameters (Å^2^)

**Table:**

	*x*	*y*	*z*	*U*_iso_*/*U*_eq_	
N1	−0.3021 (3)	0.65494 (9)	0.12504 (7)	0.0448 (4)	
N2	1.1856 (3)	0.05066 (10)	0.59878 (8)	0.0536 (5)	
N3	0.7464 (3)	0.29359 (9)	0.24722 (7)	0.0469 (4)	
C1	−0.4416 (4)	0.59659 (12)	0.13700 (9)	0.0436 (5)	
C2	−0.3757 (3)	0.54235 (11)	0.18034 (9)	0.0384 (5)	
C3	−0.1522 (3)	0.54509 (10)	0.21419 (8)	0.0343 (5)	
C4	−0.0079 (3)	0.60627 (11)	0.20284 (8)	0.0376 (5)	
C5	−0.0885 (4)	0.65812 (12)	0.15892 (9)	0.0433 (5)	
C6	−0.0763 (4)	0.48508 (11)	0.25731 (9)	0.0385 (5)	
C7	0.1333 (4)	0.47926 (11)	0.29196 (8)	0.0385 (5)	
C8	0.2023 (4)	0.41530 (11)	0.32953 (8)	0.0381 (5)	
C9	0.4162 (4)	0.40603 (11)	0.36246 (8)	0.0389 (5)	
C10	0.4835 (4)	0.33966 (11)	0.39705 (8)	0.0379 (5)	
C11	0.6999 (4)	0.32671 (11)	0.42754 (8)	0.0386 (5)	
C12	0.7613 (4)	0.25807 (11)	0.45946 (8)	0.0375 (5)	
C13	0.9763 (4)	0.24363 (11)	0.48963 (8)	0.0383 (5)	
C14	1.0455 (3)	0.17596 (10)	0.52440 (8)	0.0346 (5)	
C15	0.8974 (4)	0.11369 (11)	0.52742 (9)	0.0418 (5)	
C16	0.9740 (4)	0.05425 (12)	0.56460 (10)	0.0480 (6)	
C17	1.3282 (4)	0.11009 (13)	0.59511 (10)	0.0526 (6)	
C18	1.2671 (3)	0.17204 (12)	0.55905 (9)	0.0436 (5)	
C19	0.5479 (4)	0.25131 (12)	0.24915 (9)	0.0444 (5)	
C20	0.4693 (3)	0.20009 (11)	0.20450 (9)	0.0391 (5)	
C21	0.5991 (3)	0.18927 (10)	0.15268 (8)	0.0346 (5)	
C22	0.8084 (3)	0.23195 (11)	0.15099 (9)	0.0415 (5)	
C23	0.8709 (4)	0.28191 (12)	0.19777 (10)	0.0459 (5)	
C24	0.5221 (3)	0.14039 (11)	0.10062 (9)	0.0388 (5)	
C25	0.3179 (4)	0.10021 (10)	0.09310 (9)	0.0388 (5)	
C26	0.2444 (4)	0.05851 (11)	0.03763 (9)	0.0409 (5)	
C27	0.0375 (4)	0.01998 (10)	0.02796 (8)	0.0410 (5)	
H1	−0.601 (3)	0.5947 (10)	0.1124 (7)	0.049*	
H2	−0.481 (3)	0.5029 (10)	0.1877 (8)	0.049*	
H4	0.151 (3)	0.6145 (9)	0.2237 (7)	0.049*	
H5	0.016 (3)	0.6982 (10)	0.1492 (7)	0.049*	
H6	−0.194 (3)	0.4458 (10)	0.2585 (8)	0.049*	
H7	0.258 (3)	0.5183 (10)	0.2906 (7)	0.049*	
H8	0.077 (3)	0.3763 (10)	0.3290 (7)	0.049*	
H9	0.539 (3)	0.4467 (10)	0.3614 (7)	0.049*	
H10	0.361 (3)	0.3004 (10)	0.3966 (7)	0.049*	
H11	0.826 (3)	0.3624 (10)	0.4272 (8)	0.049*	
H12	0.633 (3)	0.2204 (10)	0.4577 (7)	0.049*	
H13	1.103 (3)	0.2809 (10)	0.4894 (7)	0.049*	
H15	0.739 (3)	0.1129 (10)	0.5057 (8)	0.049*	
H16	0.864 (3)	0.0118 (10)	0.5671 (8)	0.049*	
H17	1.483 (3)	0.1070 (10)	0.6207 (8)	0.049*	
H18	1.381 (3)	0.2120 (10)	0.5576 (7)	0.049*	
H19	0.457 (3)	0.2596 (9)	0.2858 (7)	0.049*	
H20	0.319 (3)	0.1734 (10)	0.2092 (7)	0.049*	
H22	0.903 (3)	0.2249 (9)	0.1158 (7)	0.049*	
H23	1.019 (3)	0.3124 (10)	0.1963 (7)	0.049*	
H24	0.628 (3)	0.1390 (9)	0.0662 (8)	0.049*	
H25	0.207 (3)	0.1001 (9)	0.1263 (7)	0.049*	
H26	0.355 (3)	0.0600 (9)	0.0042 (8)	0.049*	
H27	−0.071 (3)	0.0190 (9)	0.0619 (7)	0.049*	

### Atomic displacement parameters (Å^2^)

**Table:**

	*U*^11^	*U*^22^	*U*^33^	*U*^12^	*U*^13^	*U*^23^
N1	0.0449 (11)	0.0430 (12)	0.0459 (10)	0.0082 (9)	0.0007 (9)	0.0022 (8)
N2	0.0463 (11)	0.0473 (12)	0.0672 (13)	0.0057 (9)	0.0044 (10)	0.0136 (9)
N3	0.0500 (11)	0.0411 (11)	0.0501 (11)	−0.0068 (9)	0.0065 (9)	−0.0035 (8)
C1	0.0402 (13)	0.0470 (15)	0.0424 (13)	0.0078 (11)	−0.0024 (10)	−0.0058 (11)
C2	0.0378 (12)	0.0369 (13)	0.0405 (12)	−0.0004 (9)	0.0029 (10)	−0.0054 (10)
C3	0.0378 (11)	0.0324 (12)	0.0330 (11)	0.0023 (9)	0.0049 (9)	−0.0040 (9)
C4	0.0335 (11)	0.0393 (13)	0.0400 (12)	−0.0003 (10)	0.0028 (9)	0.0024 (10)
C5	0.0424 (13)	0.0398 (14)	0.0480 (13)	0.0016 (10)	0.0059 (11)	0.0071 (11)
C6	0.0437 (13)	0.0315 (13)	0.0401 (12)	−0.0002 (9)	0.0029 (10)	−0.0028 (9)
C7	0.0488 (13)	0.0311 (13)	0.0358 (12)	−0.0022 (9)	0.0034 (10)	0.0012 (9)
C8	0.0476 (13)	0.0310 (12)	0.0357 (11)	0.0008 (9)	0.0040 (10)	0.0001 (9)
C9	0.0520 (13)	0.0308 (13)	0.0336 (11)	−0.0021 (10)	0.0022 (10)	−0.0011 (9)
C10	0.0508 (14)	0.0306 (13)	0.0321 (11)	−0.0005 (9)	0.0025 (10)	−0.0023 (9)
C11	0.0504 (14)	0.0326 (13)	0.0330 (11)	−0.0035 (10)	0.0033 (10)	−0.0007 (9)
C12	0.0480 (13)	0.0311 (13)	0.0335 (11)	0.0002 (9)	0.0033 (10)	−0.0022 (9)
C13	0.0449 (13)	0.0324 (13)	0.0373 (11)	−0.0021 (9)	0.0023 (10)	−0.0011 (9)
C14	0.0358 (11)	0.0353 (12)	0.0333 (11)	0.0026 (9)	0.0061 (9)	−0.0032 (9)
C15	0.0415 (12)	0.0382 (13)	0.0454 (13)	−0.0001 (10)	0.0022 (10)	0.0002 (10)
C16	0.0471 (14)	0.0398 (14)	0.0572 (14)	−0.0024 (11)	0.0062 (11)	0.0082 (11)
C17	0.0419 (14)	0.0562 (17)	0.0590 (15)	0.0076 (12)	0.0009 (11)	0.0135 (12)
C18	0.0401 (13)	0.0400 (14)	0.0505 (13)	−0.0019 (10)	0.0030 (11)	0.0037 (11)
C19	0.0505 (14)	0.0421 (14)	0.0416 (13)	−0.0030 (11)	0.0100 (10)	−0.0022 (10)
C20	0.0375 (12)	0.0356 (13)	0.0444 (12)	−0.0053 (9)	0.0050 (10)	0.0029 (10)
C21	0.0400 (11)	0.0290 (12)	0.0345 (11)	0.0022 (9)	0.0022 (9)	0.0049 (9)
C22	0.0436 (13)	0.0411 (14)	0.0408 (12)	−0.0033 (10)	0.0082 (10)	0.0017 (10)
C23	0.0424 (13)	0.0440 (14)	0.0514 (14)	−0.0091 (10)	0.0039 (11)	0.0035 (11)
C24	0.0457 (13)	0.0344 (12)	0.0372 (12)	0.0035 (10)	0.0090 (10)	0.0019 (9)
C25	0.0463 (13)	0.0298 (12)	0.0407 (13)	0.0033 (10)	0.0053 (10)	0.0005 (10)
C26	0.0510 (14)	0.0332 (13)	0.0386 (13)	0.0036 (10)	0.0045 (10)	0.0014 (10)
C27	0.0510 (14)	0.0309 (12)	0.0409 (13)	0.0030 (10)	0.0031 (10)	0.0015 (9)

### Geometric parameters (Å, °)

**Table:**

N1—C1	1.341 (2)	C12—H12	0.982 (17)
N1—C5	1.341 (2)	C13—C14	1.462 (2)
N2—C17	1.335 (2)	C13—H13	0.972 (17)
N2—C16	1.335 (2)	C14—C18	1.387 (2)
N3—C23	1.334 (2)	C14—C15	1.393 (2)
N3—C19	1.342 (2)	C15—C16	1.380 (3)
C1—C2	1.377 (3)	C15—H15	0.962 (16)
C1—H1	0.996 (16)	C16—H16	0.981 (17)
C2—C3	1.387 (2)	C17—C18	1.382 (3)
C2—H2	0.942 (17)	C17—H17	0.984 (16)
C3—C4	1.394 (2)	C18—H18	0.960 (17)
C3—C6	1.461 (3)	C19—C20	1.375 (3)
C4—C5	1.375 (2)	C19—H19	0.983 (16)
C4—H4	0.968 (16)	C20—C21	1.392 (2)
C5—H5	0.960 (17)	C20—H20	0.974 (17)
C6—C7	1.334 (2)	C21—C22	1.396 (2)
C6—H6	0.964 (17)	C21—C24	1.460 (2)
C7—C8	1.438 (2)	C22—C23	1.372 (3)
C7—H7	0.986 (17)	C22—H22	0.966 (16)
C8—C9	1.343 (2)	C23—H23	0.990 (17)
C8—H8	0.987 (17)	C24—C25	1.343 (2)
C9—C10	1.438 (3)	C24—H24	0.985 (16)
C9—H9	1.000 (17)	C25—C26	1.440 (2)
C10—C11	1.341 (2)	C25—H25	0.983 (16)
C10—H10	0.981 (17)	C26—C27	1.343 (2)
C11—C12	1.438 (2)	C26—H26	0.986 (16)
C11—H11	0.951 (17)	C27—C27^i^	1.434 (4)
C12—C13	1.337 (2)	C27—H27	0.985 (16)
			
C1—N1—C5	115.34 (17)	C18—C14—C15	115.83 (19)
C17—N2—C16	115.36 (19)	C18—C14—C13	120.08 (18)
C23—N3—C19	114.71 (18)	C15—C14—C13	124.05 (17)
N1—C1—C2	123.89 (19)	C16—C15—C14	119.54 (19)
N1—C1—H1	115.4 (10)	C16—C15—H15	119.8 (11)
C2—C1—H1	120.7 (10)	C14—C15—H15	120.6 (11)
C1—C2—C3	120.4 (2)	N2—C16—C15	124.9 (2)
C1—C2—H2	120.7 (11)	N2—C16—H16	117.1 (10)
C3—C2—H2	118.8 (11)	C15—C16—H16	118.0 (10)
C2—C3—C4	116.02 (18)	N2—C17—C18	123.9 (2)
C2—C3—C6	120.39 (18)	N2—C17—H17	114.7 (11)
C4—C3—C6	123.57 (17)	C18—C17—H17	121.5 (11)
C5—C4—C3	119.67 (18)	C17—C18—C14	120.5 (2)
C5—C4—H4	116.6 (10)	C17—C18—H18	119.2 (10)
C3—C4—H4	123.7 (10)	C14—C18—H18	120.3 (10)
N1—C5—C4	124.6 (2)	N3—C19—C20	124.76 (19)
N1—C5—H5	116.0 (10)	N3—C19—H19	114.6 (10)
C4—C5—H5	119.2 (10)	C20—C19—H19	120.6 (10)
C7—C6—C3	127.26 (19)	C19—C20—C21	119.91 (18)
C7—C6—H6	119.5 (10)	C19—C20—H20	118.8 (10)
C3—C6—H6	113.3 (10)	C21—C20—H20	121.3 (10)
C6—C7—C8	123.8 (2)	C20—C21—C22	115.66 (18)
C6—C7—H7	120.6 (10)	C20—C21—C24	124.18 (18)
C8—C7—H7	115.5 (10)	C22—C21—C24	120.07 (17)
C9—C8—C7	125.36 (19)	C23—C22—C21	119.98 (19)
C9—C8—H8	120.8 (10)	C23—C22—H22	122.8 (10)
C7—C8—H8	113.8 (10)	C21—C22—H22	117.2 (10)
C8—C9—C10	123.8 (2)	N3—C23—C22	124.97 (19)
C8—C9—H9	118.2 (10)	N3—C23—H23	115.3 (10)
C10—C9—H9	117.9 (10)	C22—C23—H23	119.8 (10)
C11—C10—C9	125.5 (2)	C25—C24—C21	127.29 (18)
C11—C10—H10	118.3 (10)	C25—C24—H24	116.6 (10)
C9—C10—H10	116.1 (10)	C21—C24—H24	116.0 (10)
C10—C11—C12	123.34 (19)	C24—C25—C26	123.86 (19)
C10—C11—H11	120.8 (11)	C24—C25—H25	119.3 (10)
C12—C11—H11	115.8 (11)	C26—C25—H25	116.8 (10)
C13—C12—C11	124.4 (2)	C27—C26—C25	125.16 (19)
C13—C12—H12	120.2 (10)	C27—C26—H26	118.6 (10)
C11—C12—H12	115.4 (10)	C25—C26—H26	116.3 (10)
C12—C13—C14	126.60 (19)	C26—C27—C27^i^	124.9 (3)
C12—C13—H13	118.8 (10)	C26—C27—H27	117.6 (10)
C14—C13—H13	114.6 (10)	C27^i^—C27—H27	117.5 (10)
			
C5—N1—C1—C2	−0.4 (3)	C13—C14—C15—C16	−176.12 (18)
N1—C1—C2—C3	−1.0 (3)	C17—N2—C16—C15	−0.3 (3)
C1—C2—C3—C4	2.1 (3)	C14—C15—C16—N2	−0.6 (3)
C1—C2—C3—C6	−176.14 (17)	C16—N2—C17—C18	0.1 (3)
C2—C3—C4—C5	−1.7 (3)	N2—C17—C18—C14	0.9 (3)
C6—C3—C4—C5	176.43 (18)	C15—C14—C18—C17	−1.7 (3)
C1—N1—C5—C4	0.8 (3)	C13—C14—C18—C17	176.04 (18)
C3—C4—C5—N1	0.3 (3)	C23—N3—C19—C20	−1.0 (3)
C2—C3—C6—C7	178.71 (19)	N3—C19—C20—C21	0.1 (3)
C4—C3—C6—C7	0.7 (3)	C19—C20—C21—C22	1.2 (3)
C3—C6—C7—C8	−174.69 (18)	C19—C20—C21—C24	−175.40 (18)
C6—C7—C8—C9	176.5 (2)	C20—C21—C22—C23	−1.6 (3)
C7—C8—C9—C10	−176.75 (17)	C24—C21—C22—C23	175.19 (17)
C8—C9—C10—C11	176.03 (19)	C19—N3—C23—C22	0.6 (3)
C9—C10—C11—C12	−177.47 (17)	C21—C22—C23—N3	0.7 (3)
C10—C11—C12—C13	179.32 (19)	C20—C21—C24—C25	1.7 (3)
C11—C12—C13—C14	177.90 (17)	C22—C21—C24—C25	−174.80 (18)
C12—C13—C14—C18	−172.99 (19)	C21—C24—C25—C26	174.09 (18)
C12—C13—C14—C15	4.5 (3)	C24—C25—C26—C27	−177.56 (19)
C18—C14—C15—C16	1.5 (3)	C25—C26—C27—C27^i^	178.4 (2)

Symmetry codes: (i) −*x*, −*y*, −*z*.

## References

[bb1] Bader, M. M. (2009). *Acta Cryst.* E**65**, o2006. 10.1107/S1600536809029092 PMC2977225 21583677

[bb2] Barbour, L. J. (2001). *J. Supramol. Chem.* **1** 189–191.

[bb3] Bruker (2001). *SADABS*, *SMART* and *SAINT* Bruker AXS Inc., Madison, Wisconsin, USA.

[bb4] Farrugia, L. J. (1997). *J. Appl. Cryst.* **30**, 565.

[bb5] Farrugia, L. J. (1999). *J. Appl. Cryst.* **32**, 837–838.

[bb6] Gao, X., Friscic, T. & MacGillivray, L. R. (2004). *Angew. Chem. Int. Ed.* **43**, 232–36. 10.1002/anie.200352713 14695618

[bb7] Sheldrick, G. M. (2008). *Acta Cryst.* A**64**, 112–122. 10.1107/S0108767307043930 18156677

[bb8] Spek, A. L. (2009). *Acta Cryst.* D**65**, 148–155. 10.1107/S090744490804362X PMC2631630 19171970

[bb9] Woitellier, S., Launay, J. P. & Spangler, C. W. (1989). *Inorg. Chem.* **28**, 758–762.

